# Supplementary material for: Co-integrate Col3m bla NDM-1-harboring plasmids in clinical Providencia rettgeri isolates from Argentina
Source: Microbiol Spectr. 2023 Sep 21;11(5):e01651-23. doi: 10.1128/spectrum.01651-23 (PMC10581215; doi:10.1128/spectrum.01651-23)
Supplement: Supplemental material — Tables S1 and S2; Fig. S1 and S2. [file spectrum.01651-23-s0001.pdf]

TABLE S1. Primers used to confirm the β-lactamase gene.

Target[a]	Primers	Sequence (5'-3')	Reference
bla$_{CTX-M}$	CTX-Mu F	ATG TGC AGY ACC AGT AAR GT	Pagani, et al, JCM 41(9):4264-4269, 2003
	CTX-Mu R	TGG GTR AAR TAR GTS ACC AGA	
bla$_{IMP}$	IMP-R	CCG CCT TGT TAG AAA TTA G	NRRLAR
	IMP-F	TCG TTT GAA GAA GTT AAC G	
bla$_{NDM}$	NDM-F	AGC ACA CTT CCT ATC TCG AC	NRRLAR
	NDM-R	GGC GTA GTG CTC AGT GTC	
bla$_{OXA}$	O48/54-F	ATG CGT GTA TTA GCC TTA TCG G	NRRLAR
	O48-R2	TGA GCA CTT CTT TTG TGA TG	
bla$_{PER}$	PER 6 F	GCC CTG ATG ATC TGG AGC CTT	NRRLAR
	PER 1-578F	GGC CTG ACG ATC TGG AAC CTT	
	PER U1-8 R	TAA CCG CTs TGG TCC TGT GGT	
bla$_{PER-2}$	PER-2-plus	GTA GTA TCA GCC CAA TCC CC	NRRLAR
	PER-2-minus	CCA ATA AAG GCC GTC CAT CA	
bla$_{VIM}$	VIM-F	AGT GGT GAG TAT CCG ACA G	Miriagou, AAC 395, 2003
	VIM-R	GTC TCC ACG CAC TTT CAT	

[a]"Target" indicates the genes encompassed by the corresponding amplification products. NRRLAR: National and Regional Reference Laboratory in Antimicrobial Resistance

Table S2. Epidemiological information and Antimicrobial susceptibility profile

	M15628		M15758		M15793	
Hospital (province)	H1 (CABA)		H1 (CABA)		H2 (CABA)	
Isolation site (date of isolation)	Catheter(April 6th, 2013)		Urine (June 29th, 2013)		Screening (October 15th, 2013)	
Underlying disease	Diabetes with prolonged hospitalization		Prostate cancer with liver and lung metastasis		Ischemic stroke	
**Antibiotic**	**MICs (mg/L**	**Int.**	**MICs (mg/L**	**Int.**	**MICs (mg/L**	**Int.**
Imipenem[b]	8	R	8	R	16	R
Imipenem/EDTA[b,c]	1	-	0.5	-	1	-
Meropenem[b]	8	R	8	R	16	R
Meropenem/EDTA[b,c]	0.12	-	0.12	-	0.12	-
Ertapenem[b]	4	R	4	R	4	R
Aztreonam[b]	16	R	0.03	S	16	R
Cefoxitin[b]	32	R	32	R	32	R
Cefotaxime[b]	256	R	64	R	128	R
Ceftazidime[b]	>256	R	>256	R	>256	R
Cefepime[b]	64	R	16	R	32	R
Cefazolin[d]	>8	R	>8	R	>8	R
Gentamicin	1	S	1	S	≤1	S
Amikacin	2	S	2	S	≤2	S
Nalidixic acid	>16	R	>16	R	≥32	R
Ciprofloxacin	>2	R	>2	R	≥4	R
Levofloxacin[d]	>2	R	>2	R	>2	R
Trimethoprim/sulfamethoxazole	>16	R	>16	R	≥320	R
Fosfomicyn[e]	≤16	S	≤16	S	≤16	S
Ampicillin/sulbactam	>16	R	>16	R	≥32	R
Piperacillin/tazobactam	>64	R	64	I	≥128	R

CABA: Capital district, Int., interpretation; R: resistant; S, susceptible; I, intermediate.

MIC, minimum inhibitory concentration; EDTA, ethylene diamine tetra-acetic acid.

[a] Antimicrobial susceptibility testing and interpretation criteria according to Clinical and Laboratory Standards Institute (CLSI) standards (M100-S24).

[b] MICs were determined using agar dilution; MICs of other antibiotics were determined using the VITEK1 2C (AST-N082 card) (bioMérieux, Lyon, France).

[c] EDTA at a fixed concentration of 0.4 mM.

[d]MICs were determined by the automatized Phoenix (BD)

[e]MIC interpretation criteria according to EUCAST.

Figure S1.

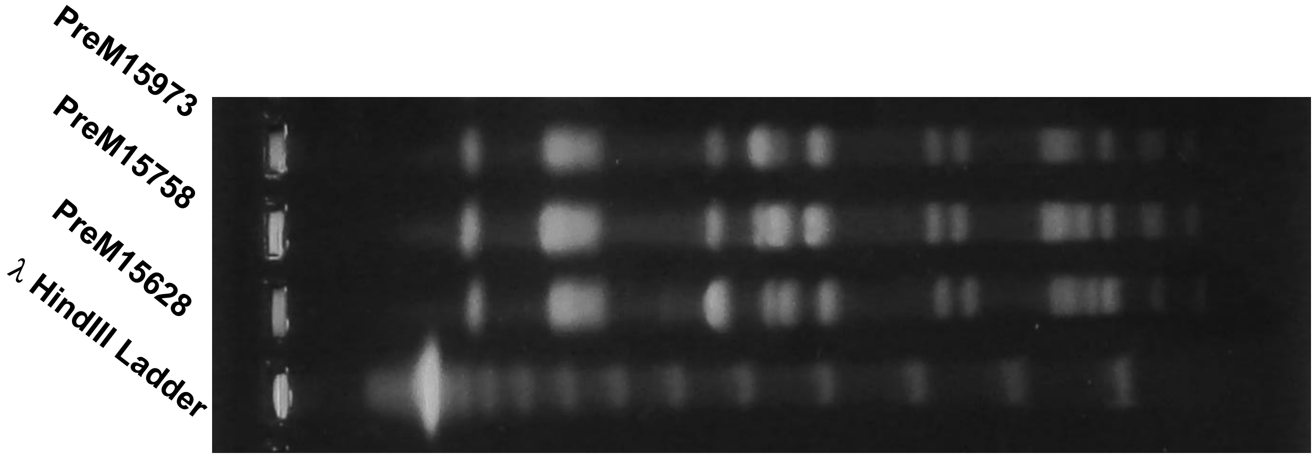

N*ot*I macrorestriction pattern of *P. rettgeri* clinical isolates obtained by PFGE. The band pattern was analyzed according to Tenover criteria J Clin Microbiol. 1995 Sep; 33(9): 2233–2239. doi: 10.1128/jcm.33.9.2233-2239.1995

Figure S2A

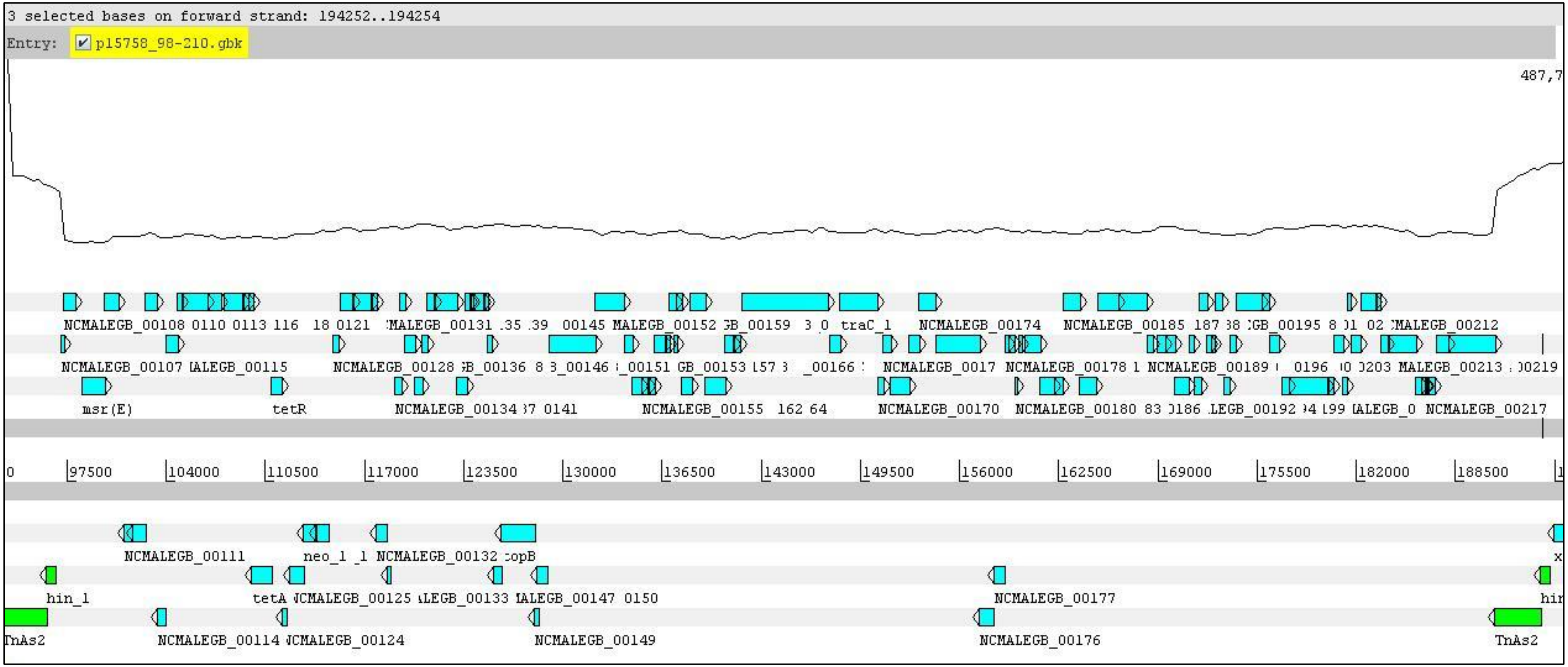

Figure S2A. Screenshot of the Artemis mapping of PreM15758 long reads against the *in silico* concatenated fasta sequence of p15758C_98 into p15758B_210 (p15758_98-210). Above, the thin black line indicates depth of coverage. Orfs are represented as arrows in their transcription orientation in light blue. The boundaries of the 98 Kb element is evidenced by the depth of coverage line. In green, hin-TnAs2, the putative homologous recombination site flanking the 98kb element .

Figure S2B

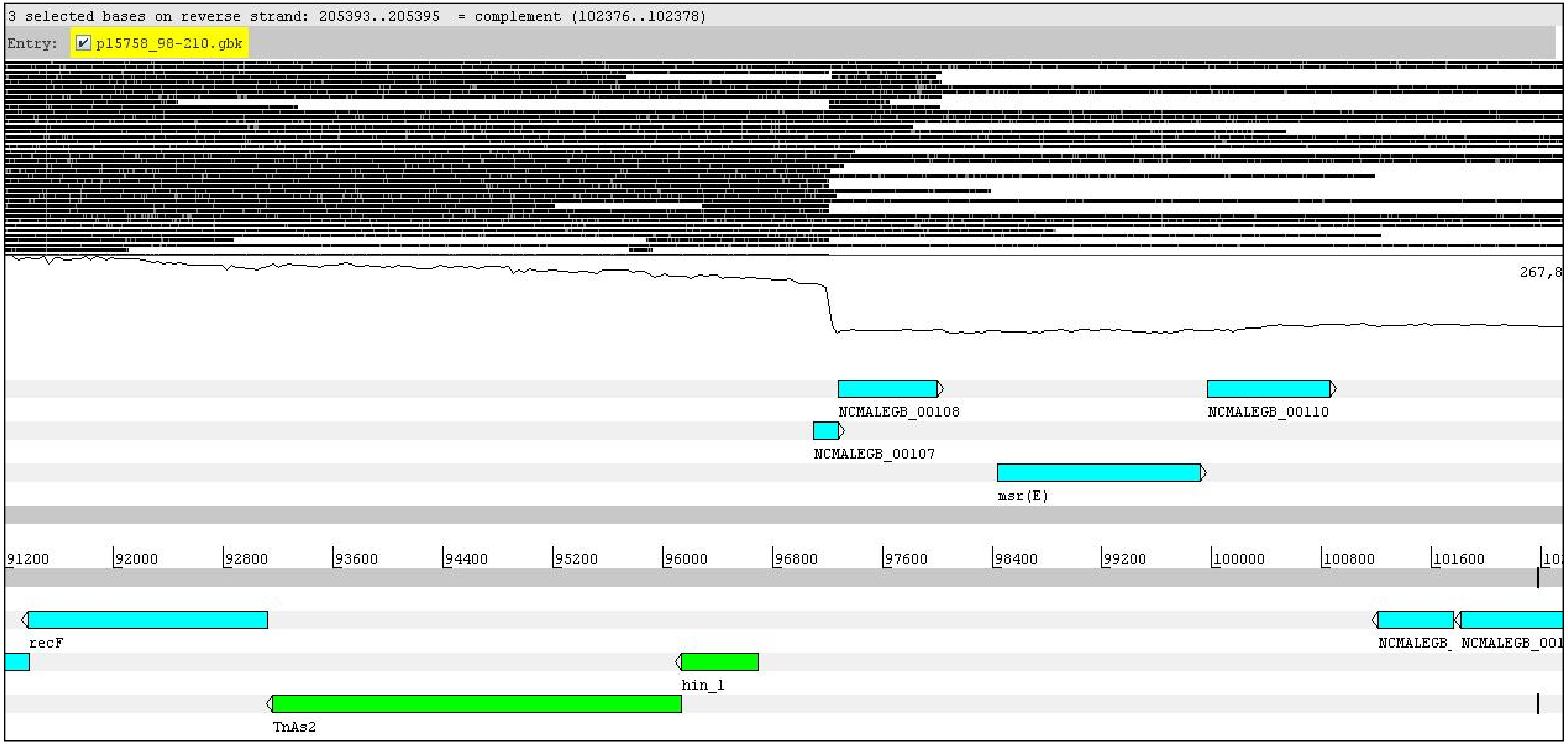

Figure S2B. Closer screenshot view of the mapping PreM15758 long reads against the *in silico* concatenated fasta sequence of p15758C_98 into p15758B_210 (p15758_98-210). The upper box shows the reads mapped. Below the reads, thin black line shows depth of coverage. Orfs are represented as arrows in their transcription orientation in light blue. The boundary of the 98 Kb element is evidenced by the reads and the depth of coverage. In green, hin-TnAs2, the putative homologous recombination site.
